# Molecular evolution of the Bovini tribe (Bovidae, Bovinae): Is there evidence of rapid evolution or reduced selective constraint in Domestic cattle?

**DOI:** 10.1186/1471-2164-10-179

**Published:** 2009-04-24

**Authors:** Sean MacEachern, John McEwan, Alan McCulloch, Andrew Mather, Keith Savin, Mike Goddard

**Affiliations:** 1 Primary Industries Research Victoria, Animal Genetics and Genomics, Attwood, VIC 3049, Australia; 2 Latrobe University, Department of Genetics, Bundoora, VIC 3086, Australia; 3 Animal Genomics, AgResearch, Invermay, Private Bag 50034, Mosgiel, New Zealand; 4 Melbourne University, School of Agriculture and Food Systems, Melbourne VIC 3000, Australia; 5 Avian Disease and Oncology Laboratory 3606 E Mt Hope Rd, East Lansing, MI 48823, USA

## Abstract

**Background:**

If mutation within the coding region of the genome is largely not adaptive, the ratio of nonsynonymous (dN) to synonymous substitutions (dS) per site (dN/dS) should be approximately equal among closely related species. Furthermore, dN/dS in divergence between species should be equivalent to dN/dS in polymorphisms. This hypothesis is of particular interest in closely related members of the Bovini tribe, because domestication has promoted rapid phenotypic divergence through strong artificial selection of some species while others remain undomesticated. We examined a number of genes that may be involved in milk production in Domestic cattle and a number of their wild relatives for evidence that domestication had affected molecular evolution. Elevated rates of dN/dS were further queried to determine if they were the result of positive selection, low effective population size (N_e_) or reduced selective constraint.

**Results:**

We have found that the domestication process has contributed to higher dN/dS ratios in cattle, especially in the lineages leading to the Domestic cow (*Bos taurus*) and Mithan (*Bos frontalis*) and within some breeds of Domestic cow. However, the high rates of dN/dS polymorphism within *B. taurus *when compared to species divergence suggest that positive selection has not elevated evolutionary rates in these genes. Likewise, the low rate of dN/dS in Bison, which has undergone a recent population bottleneck, indicates a reduction in population size alone is not responsible for these observations.

**Conclusion:**

The effect of selection depends on effective population size and the selection coefficient (N_e_s). Typically under domestication both selection pressure for traits important in fitness in the wild and N_e _are reduced. Therefore, reduced selective constraint could be responsible for the observed elevated evolutionary ratios in domesticated species, especially in *B. taurus *and *B. frontalis*, which have the highest dN/dS in the Bovini. This may have important implications for tests of selection such as the McDonald-Kreitman test. Surprisingly we have also detected a significant difference in the supposed neutral substitution rate between synonymous and noncoding sites in the Bovine genome, with a 30% higher rate of substitution at synonymous sites. This is due, at least in part, to an excess of the highly mutable CpG dinucleotides at synonymous sites, which will have implications for time of divergence estimates from molecular data.

## Background

Domestication implies a period of intense phenotypic selection that should result in dramatic changes to specific areas of the genome. Therefore, one might expect to see a signature of selection in the DNA sequence of domestic species as measured by the ratio of nonsynonymous to synonymous substitutions per site (dN/dS) [1].

The McDonald-Kreitman test [2] compares the dN/dS rate in the divergence between species to the dN/dS rate in common polymorphisms on the assumption that the latter represents the rate in neutral mutations. In this paper we extend previous findings [3] by comparing the dN/dS rate among 14 closely related species of bovid with the dN/dS rate in polymorphisms from *B. taurus*. Another neutral estimate of dN/dS mutations [3] has been developed that is based on ancestral polymorphisms in coding regions that have undergone lineage sorting which were found to be neutral. We compare the dN/dS rate at these aberrant sites with that observed between species and breeds of bovid. Specifically we wish to test the hypothesis that domestication increases dN/dS either in general, or at least in some genes.

The Bovinae subfamily is a useful group in which to study molecular evolution as it encompasses several domestic species and a number of wild relatives that have experienced very little or no domestication, which all diverged from a common ancestor ~10–15MYA. Table 1 presents a summary of the animals in our study; their classification and whether they have been subject to large scale domestication. Of the Bovinae representatives, Domestic cattle (*Bos taurus*) are amongst the most developed by domestication and artificial (positive) selection. Archaeological and genetic data suggest the domestication of *B. taurus *occurred approximately ten thousand years ago and that it was limited to just a few events in the Fertile Crescent and North Africa [4-6]. Since that time domesticated cattle have experienced various selective pressures that have resulted in major phenotypic shifts for a range of traits. For example, selection for better lactation ability and persistence, primarily over the past 5,000 years, has increased milk production [1,7]. This selection has increased dramatically for dairy breeds like Holstein over the last 50 years with the use of artificial insemination, all of which have contributed to a large increase in cattle numbers and a significant decrease in effective population size [8]. Thus, genes suspected of being involved with increased milk production in *B. taurus *should be good targets for identifying positive selection that may have resulted from domestication.

**Table 1:** Summary of the representative species and whether they were considered domesticated

Subfamily Bovinae			
Tribe	Representative species	Domesticated	Note

Tragelaphini	*Taurotragus oryx* (Eland)	No	Undomesticated
Bovini	*Syncerus caffer* (African buffalo)	No	Undomesticated
Bovini	*Bubalus bubalis* (Water buffalo)	Yes	Domesticated in India and Asia
Bovini	*Bubalus carabensis* (Swamp buffalo)	Yes	Domesticated in East Asia
Bovini	*Bison bison* (Bison)	No	Undomesticated
Bovini	*Bos grunniens* (Yak)	Yes/No	Domesticated in Asia & some wild species still exist
Bovini	*Bos taurus* (Domestic cow)	Yes	Domesticated in Mideast
Bovini	*Bos javanicus* (Bali cattle)	Yes/No	Domesticated in Indonesia & some wild species still exist
Bovini	*Bos gaurus* (Gaur)	No	Some history, but in general considered undomesticated
Bovini	*Bos frontalis* (Mithan)	Yes	May be a domestic version of *B. gaurus*

Because of the cost of sequencing, only a limited number of genes could be analysed from genomic DNA. Therefore, a candidate gene approach was used to identify genes that may be involved in milk production. In this study genes that underlie putative QTL in Holstein for milk production traits from 20 centiMorgan (cM) regions on Bovine chromosomes 1, 2, 6, 9 and 26 were examined [9-11] and hence should give a representative look at a wide sample of genes from the cattle genome. Additional genes were examined from previous work on lactation-associated genes with high expression in the mammary gland and relatively rapid rates of evolution for pairwise comparisons between human and cattle [12]. These genes were mapped to regions of Bovine chromosomes 14 and 21 and did not underlie QTL. A more detailed description of the genes, species sampled and their phylogenetic relationships have been described previously [3].

In the present study, genomic DNA variation is analysed from 15 autosomal genes within and between 14 closely related representatives of the Bovinae subfamily in an effort to determine if selective or demographic processes (eg changes in effective population size) governed molecular evolution in Domestic cattle and whether the domestication process was associated with increased rates of molecular evolution. Therefore, dN/dS ratios calculated for divergence, polymorphism and ancestral polymorphisms were used to identify rapid molecular evolution in specific lineages, genes and in domestic vs undomesticated animals.

## Results

### Differences between genes and species in the rate of evolution from ancestral to extant species

We sequenced 84 amplicons from 15 autosomal genes in 14 representatives of the Bovinae subfamily resulting in ~52,800 base pairs of sequence in each species, which we have submitted to Genbank [3]. The sequence of the common ancestor was inferred and used to count the synonymous and nonsynonymous substitutions between the ancestral sequence and each extant species. An analysis of variance tested the significance of differences in the rate of evolution on the 14 branches of the evolutionary tree (analysis GLM1 in Methods). Table 2 shows a summary of the p-values from the analysis of variance. Pairwise comparisons between the ancient and contemporary samples in GLM 1 yielded significant (p < 0.05) effects for amplicon (a) for all of the variables tested. However, difficulties arise with this analysis due to the lack of independence for substitutions between lineages. Because some mutations have occurred in a common co-ancestor, these mutations are being considered more than once causing the F-test to be anti-conservative. This problem does not affect the F-test for gene because it compares gene and amplicon within gene mean squares (see Methods). That is, we test if the difference between genes is greater than the variation between amplicons within genes. Significant gene effects were detected for dN and dN/(dN+dS). Thus, different genes have accumulated different numbers of amino acid changing mutations, which may be due to different levels of selective constraint. Table 3 presents the estimated effects for GLM 1 examining the variation in genes for dN and dN/(dN+dS). Amongst the most rapidly evolving genes are 5HT1F and EGF, while the slowest evolving genes are IGFBP2 and IGFBP5.

**Table 2:** Significance tests for the effect of gene (g), amplicon within gene (a) and species (s).

Variables	g	a	s	e
dN	***	***	0.777	0.00001
dS	0.813	***	***	0.0005
dN/(dN+dS)	***	***	0.287	0.0309
Dr	0.636	***	0.251	0.0127
Dc	***	***	0.413	0.1942
Dr/(Dr+Dc)	0.986	***	0.296	0.0138
dI	0.637	***	***	0.00006

Numbers in columns g, a and s are p-values, *** = p < 0.001

The error MS is given in the final column (e)

**Table 3:** Estimated gene effects in bold (and s.e.) are deviations from mean values from GLM 1 minus amplicon y = u + g + s + e effect for dN and dN/(dN+dS), where g and s = gene and species effects, respectively and e = residual error.

Estimated effect	dN	dN/(dN+dS)
Mean (μ)	**0.00144 **(0.001)	**0.5844 **(0.077)
Gene (g)		
5HT1F	**0.0**	**0.0**
5HT6	**0.00103 **(0.0015)	**-0.268 **(0.192)
EGF	**0.00540 **(0.0015)	**0.438 **(0.126)
ERa	**-0.0003 **(0.0015)	**-0.465 **(0.088)
GMEB1	**-0.0015 **(0.0015)	**-0.572 **(0.086)
HFABP	**0.0005 **(0.0015)	**-0.324 **(0.115)
IGFBP2	**-0.0015 **(0.0015)	**-0.603 **(0.192)
IGFBP5	**-0.0016 **(0.0015)	**-0.577 **(0.193)
ITGBP5	**-0.0016 **(0.0015)	**-0.569 **(0.089)
LACS3	**0.00675 **(0.0015)	**-0.4371 **(0.086)
MFG8E	**-0.0007 **(0.0015)	**-0.4853 **(0.089)
NRIP1	**0.0036 **(0.0015)	**-0.3341 **(0.087)
PABPC1	**-0.0015 **(0.0015)	**-0.5685 **(0.088)
PIT1	**-0.0016 **(0.0015)	**-0.5710 **(0.087)
PRRP	**-0.0015 **(0.0015)	**0**

Surprisingly, a significant difference between species for the assumed neutral mutation rate in dS and dI was detected. The differences between species for dS were found to be restricted to the Water buffalo, which show a significantly higher rate of silent site evolution, while the breeds of *B. taurus *were amongst the lowest when compared to the ancestral sequence. The significant effect detected in species for the number of intronic substitutions per site (dI) was the result of a higher rate of substitution in the Eland lineage (results not shown). This is likely an anomaly in the ancestral sequence, as the Eland outgroup's divergence time from the ancestral sequence is not similar to other members of the Bovini tribe.

The rate of radical (Dr) to conservative mutations (Dc) was not significantly different between genes or species, because most amino acid changing mutations were conservative in nature. Therefore, Dr/Dc is not a useful measure of molecular evolution for this study.

**Figure 1:**
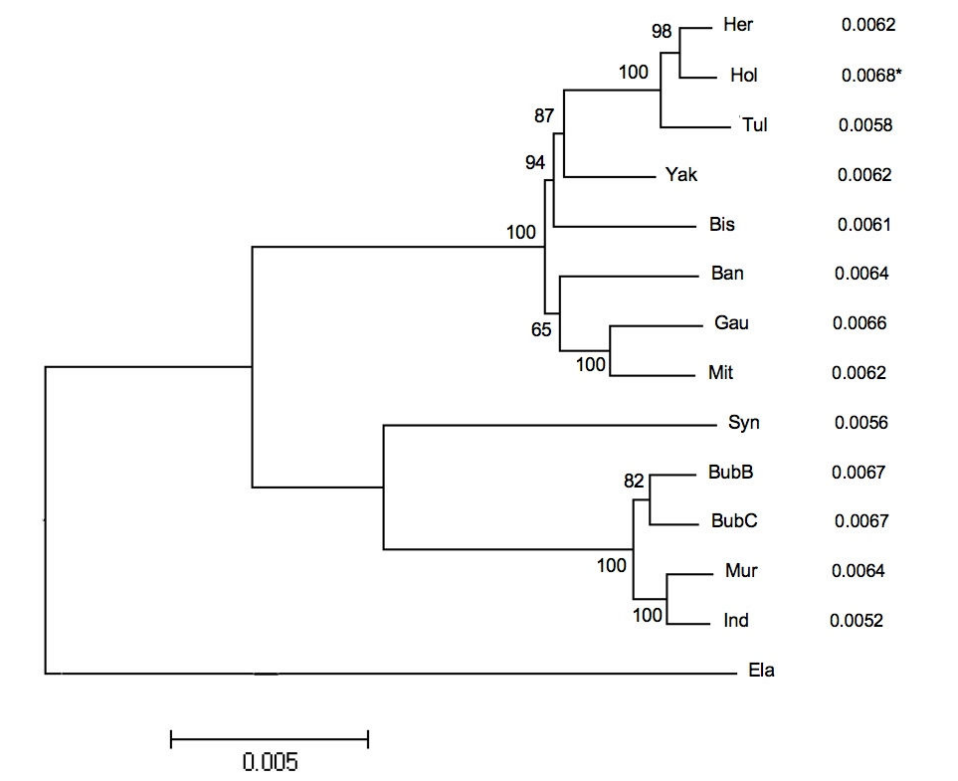
Comparison of a neutral phylogeny and amino acid variation, neighbour joining analysis for members of the Bovini tribe (Anc: Ancient, Ban: Banteng, Bis: Bison, BubB & BubC: Asian buffalo, water and swamp type, Ela: Eland, Gau: Gaur, Her: Hereford, Ind & Mur: Indian water buffalo, Mit: Mithan, Hol: Holstein, Syn: African buffalo, Tul: Tuli, and Yak) using Kimura two parameter model with bootstrap values (5000 replicates) overlying branchpoints from an alignment of 21,055 bp with 1526 segregating sites from noncoding sequence, with the corresponding number of amino acid changes per site to the right of each species when compared to the Eland outgroup * highlights the largest number of aa changes per site.

In figure 1 we present a partial phylogenetic reconstruction from over 21,000 noncoding sites and 1,500 substitutions at these sites. As can be seen in the phylogenetic tree one river buffalo (BubB) appears to be more closely related to a swamp buffalo (BubC) than to other river buffalo, which may be due to recent introgression with a swamp buffalo. Despite this anomaly, the majority of species sampled appear to be related as we expected. More details regarding the relationships between these animals can be found in MacEachern et al [3]. We also compare the neutral phylogenetic tree with the variation found at amino acids with the number of amino acid changing mutations per site (dN). The highest number of amino acid changes per site identified was in Holstein (Figure 1, 0.0068). Where a direct contrast between domestic and undomesticated animals was possible, like domestic cattle and water buffalo, the breeds under more intense artificial selection (Holstein and Hereford or Murrah, Asian water and swamp buffalo) have accumulated more amino acid changes than less intensely selected breeds (Tuli and the nondescript breed of Indian water buffalo or species like the African buffalo). However, as described in GLM 1 (Table 2) these differences between species are not quite significant. Surprisingly, the dN rate in Gaur is higher than Mithan, suggesting a faster mutation rate in this species.

The model GLM 2 (see Methods) tested the interaction between gene and species (g.s) on rates of evolution. No significant interaction was detected between gene and species for any of the dependent variables, indicating that the differences detected for genes in GLM 1 were not particular to any one species. Therefore, high substitution or evolutionary rates for a given gene were common across the entire phylogeny and the differences in evolutionary rate between genes were most likely a result of different levels of selective constraint amongst genes rather than species.

### Comparison of substitutions at synonymous and noncoding sites and CpG dinucleotides

Synonymous and noncoding sites were examined for differences in the number of substitutions per site (dS and dI, respectively). We identified a significant difference between the number of substitutions per site for dS and dI, with a significantly higher rate of substitution detected at synonymous sites. Overall a ~30% higher rate of substitution at synonymous sites was detected, which was determined to be significant (p < 0.001). An examination of whether CpG dinucleotides were over represented in substitutions at synonymous or noncoding sites uncovered a significantly higher proportion of silent substitutions at synonymous sites that involve CpG dinucleotides than at noncoding sites (Table 4, p < 0.001). The difference between synonymous and noncoding substitutions per site decreases when substitutions involving CpG dinucleotides are removed from the analysis with only a 10% higher rate of substitution at synonymous sites, however, this difference is still significant (Table 4, p < 0.001).

**Table 4:** Summary of the total number of sites, substitutions and substitutions per site at synonymous and noncoding regions, substitutions involving CpGs, relative proportion of substitutions involving CpG dinucleotides and the number of substitutions per site without the influence of CpG dinucleotides

	Total sites	Total substitutions	Substitutions per site	Substitutions involving CpGs	Proportion of substitutions involving CpGs	Substitutions per site sans CpG
Synonymous	286,995	7,394	0.026	2,262	0.31	0.018
Noncoding	3,767,771	68,602	0.018	8,212	0.12	0.016

Chi-Square comparing the relative proportion of substitutions involving CpG dinucleotides at synonymous and noncoding sites χ^2 ^= 1,300, p < 0.001 and substitutions that did not involve CpG dinucleotides χ^2 ^= 55.7, p < 0.001

### Genetic distance between species and breeds

**Figure 2:**
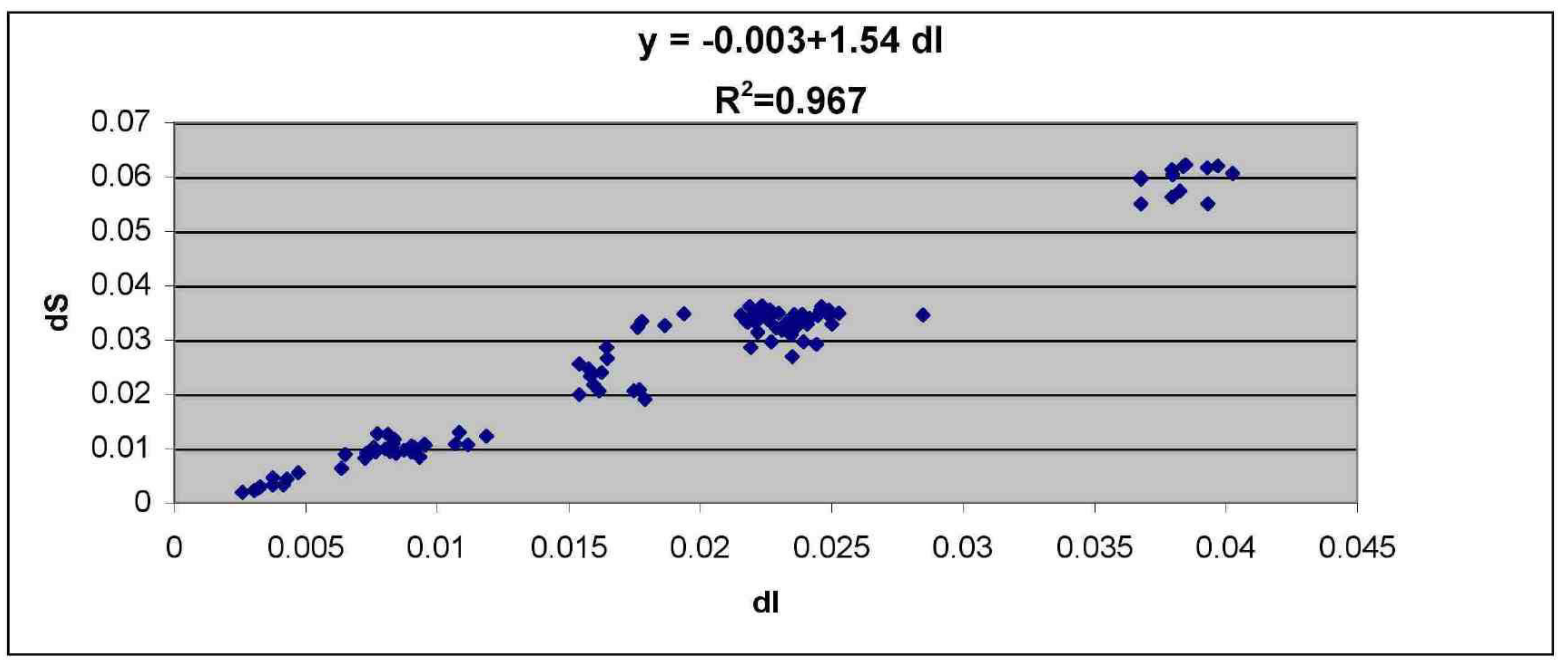
**the rate of substitution at synonymous sites (dS) plotted against the rate of substitution at noncoding sites (dI) for all paiwise comparisons within the Bovini tribe**.

Pairwise comparisons for dI and dS between all 14 Bovini representatives are given in table 5. The lowest values are for comparisons within a species (i.e. within Indian water buffaloes or within *Bos taurus*) and the highest are between Eland and other species. This finding is also represented graphically in figure 2, with a significant linear relationship identified between dS and dI for pairwise comparisons summed across all genes. The simplest explanation is that both dI and dS are largely neutral substitutions that accumulate over greater evolutionary time.

**Table 5:** Pairwise comparisons between Bovinae representatives for the number of silent substitutions per site summarised for all genes, with intronic substitutions (dI) below the diagonal and synonymous substitutions (dS) above the diagonal in bold

	Her	Hol	Tul	Bis	Yak	Ban	Mit	Gau	BubB	Mur	Ind	BubC	Syn	Ela
Her	-	**0.002**	**0.003**	**0.011**	**0.01**	**0.011**	**0.01**	**0.012**	**0.034**	**0.035**	**0.034**	**0.034**	**0.029**	**0.058**
Hol	0.003	-	**0.003**	**0.01**	**0.008**	**0.009**	**0.008**	**0.011**	**0.032**	**0.032**	**0.032**	**0.033**	**0.026**	**0.053**
Tul	0.004	0.003	-	**0.01**	**0.009**	**0.011**	**0.01**	**0.013**	**0.034**	**0.035**	**0.034**	**0.035**	**0.029**	**0.059**
Bis	0.01	0.008	0.009	-	**0.009**	**0.01**	**0.013**	**0.012**	**0.034**	**0.033**	**0.034**	**0.035**	**0.031**	**0.06**
Yak	0.008	0.007	0.007	0.006	-	**0.009**	**0.009**	**0.009**	**0.033**	**0.034**	**0.034**	**0.035**	**0.028**	**0.059**
Ban	0.011	0.009	0.01	0.008	0.008	-	**0.011**	**0.012**	**0.033**	**0.033**	**0.032**	**0.033**	**0.029**	**0.054**
Mit	0.009	0.009	0.009	0.008	0.008	0.008	-	**0.006**	**0.033**	**0.034**	**0.034**	**0.034**	**0.03**	**0.059**
Gau	0.012	0.011	0.011	0.008	0.008	0.008	0.006	-	**0.034**	**0.034**	**0.035**	**0.035**	**0.031**	**0.034**
BubB	0.024	0.023	0.023	0.022	0.022	0.023	0.022	0.023	-	**0.004**	**0.006**	**0.005**	**0.02**	**0.057**
Mur	0.025	0.024	0.025	0.024	0.023	0.024	0.024	0.025	0.024	-	**0.002**	**0.003**	**0.019**	**0.055**
Ind	0.028	0.025	0.025	0.024	0.024	0.025	0.023	0.025	0.005	0.003	-	**0.003**	**0.02**	**0.058**
BubC	0.023	0.022	0.023	0.022	0.022	0.022	0.022	0.022	0.004	0.004	0.003	-	**0.02**	**0.053**
Syn	0.024	0.024	0.024	0.022	0.022	0.023	0.023	0.023	0.017	0.018	0.018	0.016	-	**0.057**
Ela	0.04	0.039	0.039	0.038	0.038	0.038	0.04	0.038	0.038	0.038	0.038	0.037	0.037	-

Species: (Ban: Banteng, Bis: Bison, Ind & Mur: Water buffalo (river type), BubB & BubC: Water buffalo (swamp type), Ela: Eland, Gau: Gaur, Her: Hereford, Mit: Mithan, Hol: Holstein, Syn: African buffalo, Tul: Tuli, and Yak)

In table 6 the pairwise comparisons for dN/dS and dN are presented, lower and upper diagonal, respectively. The patterns of divergence for dN are similar to those found for dI and dS with the smallest values found between breeds of Domestic cattle and buffalo, while the largest values were consistently found between species. Pairwise comparisons for dN/dS show a different pattern, with some of the largest values detected between breeds of *B. taurus *and other closely related species from the Bovina subtribe. Interestingly, pairwise comparisons between buffalo breeds or species that have been separated over similar time frames often show much smaller evolutionary ratios (BubB vs BubC and Ind vs Mur)

**Table 6:** Pairwise comparisons between all Bovinae representatives for the ratio of nonsynonymous to synonymous substitutions per site (dN/dS) summarised for all genes below the diagonal and dN above the diagonal in bold

	Her	Hol	Tul	Bis	Yak	Ban	Mit	Gau	BubB	Mur	Ind	BubC	Syn	Ela
Her	-	**0.0005**	**0.0008**	**0.0013**	**0.0012**	**0.0014**	**0.0014**	**0.0012**	**0.0035**	**0.0032**	**0.0035**	**0.0034**	**0.0028**	**0.0062**
Hol	0.214	-	**0.0003**	**0.0008**	**0.0009**	**0.0009**	**0.0018**	**0.0008**	**0.0031**	**0.0029**	**0.003**	**0.0032**	**0.0023**	**0.0068**
Tul	0.239	0.112	-	**0.001**	**0.001**	**0.001**	**0.0019**	**0.0011**	**0.0029**	**0.0023**	**0.0027**	**0.0029**	**0.0023**	**0.0058**
Bis	0.12	0.085	0.099	-	**0.0011**	**0.0009**	**0.0017**	**0.0006**	**0.0031**	**0.0025**	**0.0026**	**0.003**	**0.0023**	**0.0061**
Yak	0.125	0.107	0.111	0.126	-	**0.0012**	**0.0019**	**0.0011**	**0.0036**	**0.0032**	**0.0033**	**0.0035**	**0.0025**	**0.0062**
Ban	0.126	0.096	0.093	0.089	0.127	-	**0.0018**	**0.001**	**0.0032**	**0.003**	**0.0028**	**0.0034**	**0.0024**	**0.0064**
Mit	0.185	0.211	0.185	0.132	0.21	0.163	-	**0.0011**	**0.0035**	**0.003**	**0.0033**	**0.0034**	**0.0028**	**0.006**
Gau	0.096	0.076	0.088	0.049	0.121	0.087	0.165	-	**0.0028**	**0.0028**	**0.0026**	**0.0029**	**0.0019**	**0.0066**
BubB	0.102	0.097	0.084	0.09	0.108	0.095	0.104	0.081	-	**0.0006**	**0.0006**	**0.0002**	**0.0024**	**0.0067**
Mur	0.091	0.088	0.065	0.074	0.093	0.088	0.087	0.08	0.135	-	**0.0001**	**0.0005**	**0.002**	**0.0064**
Ind	0.01	0.091	0.077	0.075	0.095	0.086	0.095	0.074	0.099	0.06	-	**0.0006**	**0.0019**	**0.0052**
BubC	0.098	0.095	0.083	0.085	0.097	0.102	0.098	0.08	0.049	0.149	0.185	-	**0.0023**	**0.0067**
Syn	0.097	0.088	0.078	0.074	0.088	0.079	0.091	0.06	0.115	0.108	0.092	0.112	-	**0.0056**
Ela	0.102	0.124	0.094	0.098	0.101	0.114	0.098	0.0106	0.113	0.111	0.086	0.122	0.094	-

Species: (Ban: Banteng, Bis: Bison, Ind & Mur: Water buffalo (river type), BubB & BubC: Water buffalo (swamp type), Ela: Eland, Gau: Gaur, Her: Hereford, Mit: Mithan, Hol: Holstein, Syn: African buffalo, Tul: Tuli, and Yak)

**Figure 3:**
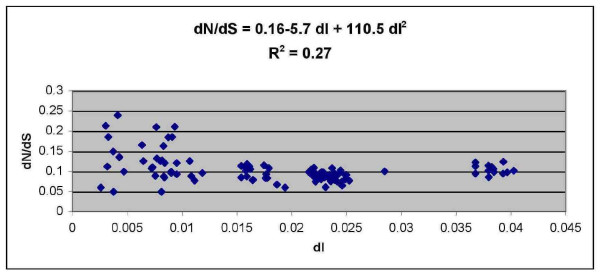
**Relationship between the evolutionary ratio (dN/dS) plotted against the number of substitutions for noncoding DNA per site (dI) for all pairwise comparisons within Bovini**.

A significantly negative relationship was found between the evolutionary ratio (dN/dS) and the number of substitutions in noncoding DNA (dI) (Figure 3). The relationship appears to be quadratic, which suggests that there is a relative abundance of dN:dS over short time scales and that negative selection is more effective at removing deleterious mutations over increasing evolutionary time. Thus, dN/dS is variable, but includes some high values when dI is small. Conversely, at high dI, dN/dS is approximately 0.1.

### Divergence, polymorphism and domestication

If selective constraint is reduced over small time scales consistently higher rates of molecular evolution should be seen between very closely related species. Likewise, if this phenomenon is common for polymorphisms the highest rates of evolution should be seen within species. Alternatively, if domestication has increased the evolutionary rate in *B. taurus *by relaxing selective constraint, higher rates of divergence between species should be seen in domesticated species when compared to their undomesticated counterparts and a higher rate of polymorphism within *B. taurus *should be found when compared to less intensely domesticated species such as the Water buffalo.

Pairwise comparisons presented in table 7 between closely related species from the Bovina subtribe have identified no large evolutionary ratios (dN/dS > 0.1) for two undomesticated animals that recently shared a common ancestor (Gaur vs Bison, Gaur vs Banteng, Bison vs Banteng). Therefore, the negative relationship detected in figure 3 may be the result of comparisons animals that have undergone a relatively intense domestication event. Pairwise comparisons, between closely related species where at least one species has a history of domestication (Table 7, bottom left) (Mithan, Yak and Domestic cattle) were generally higher than pairwise comparisons between undomesticated species. The values for dN/dS appear to increase again when two domestic animals are compared (Table 7, bottom right). In fact all dN/dS comparisons with values > 0.15 involve one or two domestic species (Table 7, bottom left and right).

**Table 7:** Pairwise comparisons between domestic and nondomestic members of the Bovina subtribe for dN/dS calculated for all mutations and sites sampled.

	Ban	Bis	Gau	Yak	Her	Mith	Hol	Tul
Ban	*							
Bis	0.089	*						
Gau	0.087	0.049	*					

Yak	0.127	0.126	0.121	*				
Her	0.126	0.12	0.096	0.125	*			
Mit	0.163	0.132	0.165	0.21	0.185	*		
Hol	0.096	0.085	0.076	0.107	0.214	0.211	*	
Tul	0.093	0.099	0.088	0.111	0.239	0.185	0.112	*

Species: (Ban: Banteng, Bis: Bison, Gau: Gaur, Her: Hereford, Mit: Mithan, Hol: Holstein, Tul: Tuli, and Yak)

Pairwise comparisons in top left between 2 undomesticated animals, bottom left between 1 domestic and one undomesticated animal, and bottom right two domestic animals

Because the Bovini tribe diverged in a star like phylogeny ~2MYA [3], comparisons between undomesticated animals (Gaur vs Bison) should be comparable to domesticated species (Yak vs Mithan). However the high rates detected between breeds of *B. taurus *could be the result of changes in the time of divergence.

To examine if selective constraint in *B. taurus *had recently been relaxed, we compared within species variation for dN/dS between Domestic cattle and buffalo. High evolutionary rates were detected (Table 6, lower diagonal) for Hereford vs Holstein and Hereford vs Tuli, with the highest ratio detected between Hereford and Tuli. In comparison, the evolutionary ratios for buffalo were generally much smaller, with Ind vs Mur showing low evolutionary ratios and BubB vs BubC showing the smallest evolutionary ratio. However, some large ratios were found in buffalo, highlighting the low accuracy of dN/dS ratios between close relatives. If intense domestication and artificial selection in *B. taurus *is affecting dN/dS rates, then dN/dS between breeds of Domestic cattle should be significantly larger than those detected between breeds of buffalo. Therefore, we have tested for significance between the ratio of nonsynonymous and synonymous substitutions within both species. None of the comparisons were significant, however, a significant difference was nearly detected for dN/dS between Hereford vs Tuli and BubB vs BubC (Table 6, lower diagonal, χ^2 ^test, p = 0.068).

### Selection tests

Holstein polymorphism data was collected from 8 animals in an effort to detect if differences existed between breeds of *B. taurus *that may be the result of recent artificial selection. Table 8 summarises the number of polymorphisms and the evolutionary rate detected in Holsteins. Only 3 nonsynonymous and 5 synonymous mutations were found in the genes 5HT1F, 5HT6 and EGF. The dN/dS ratio within Holstein is similar to that between breeds of *B. taurus*. Both are higher than dN/dS in species divergence, so a McDonald-Kreitman test gives no evidence of positive selection driving species divergence. This might be because dN/dS within *B. taurus *is higher as a result of ineffective negative or purifying selection.

**Table 8:** Summary of total Holstein polymorphisms for all genes

Noncoding sites	Ki	dI	Nonsynonymous sites	Synonymous sites	Ka	Ks	dN	dS	dN/dS
45,102	168	0.0037	8561	2671	3	5	0.00035	0.0019	0.187

### Ancient polymorphisms

A number of neutral or nearly neutral substitutions were identified from the dataset that appear to be the result of homologous mutations in a common ancestor that have subsequently been randomly assorted in modern species [3]. In total these ancestral polymorphisms corresponded to 7 nonsynonymous and 26 synonymous mutations and a dN/dS rate of 0.065.

The McDonald-Kreitman test was modified to compare dN/dS between species to dN/dS in ancient polymorphisms on the assumption that the latter are neutral. Table 9 shows the results of chi-squared tests in which Ka:Ks in a series of species differences and species polymorphisms is compared to Ka:Ks in ancient substitutions. In general, pairwise comparisons between Mithan and other members of the Bovidae have higher dN/dS than the rate in aberrant polymorphism, with significant differences detected between Mithan and Holstein cattle. This finding could be mistaken as a sign of rapid evolution in domestic cattle. However, examining the other pairwise comparisons it appears that Mithan is generating the difference with near significant results for the majority of members in the Bovidae (Table 9) and consistently large evolutionary ratios (dN/dS) in table 6 (lower diagonal).

**Table 9:** Modified McDondald-Kreitman test examining variation between ancient polymorphism and pairwise comparisons between species and breeds for the ratio of nonsynonymous (Ka) and synonymous (Ks) polymorphisms

	Ancient	Substitutions							Polymorphisms		
		Ba-Mi	Ga-Mi	He-Mi	Bi-Mi	Ho-Mi	Tu-Mi	Ya-Mi	He-Ho	He-Tu	Ho-Tu
Ka	6	17	10	18	16	17	18	19	5	8	3

Ks	27	31	18	29	36	24	29	27	7	10	8

dN/dS	0.065	0.163	0.165	0.185	0.132	0.211	0.185	0.21	0.213	0.239	0.112

χ^2^	-	2.9	2.4	3.7	1.7	4.6	3.7	4.7	2.6	4.0	0.4

p	-	0.09	0.1	0.05	0.2	0.03	0.05	0.03	0.1	0.04	0.5

Species: (Ba: Banteng, Bi: Bison, Ga: Gaur, He: Hereford, Mi: Mithan, Ho: Holstein, Tu: Tuli,, Ya: Yak)

We also applied the test to identify differences in the rate of polymorphism for *B. taurus *and *B. bubalis*. Comparisons of the polymorphisms within Domestic cattle and Water buffalo with the rate of ancient polymorphisms should help identify whether any differences in evolutionary rate can be attributed to a relaxation of selective constraint. A significant difference was detected for the number of polymorphisms between Hereford and Tuli when compared with the number of ancient polymorphisms (Table 9). The same comparison was made for polymorphisms found between both types of Water buffalo (Ind vs Mur and BubB vs BubC); however, no significant differences were detected (results not shown). Therefore, it appears that during the domestication of *B. taurus *there has been an accumulation of nonsynonymous mutations above those found at aberrant sites, which may result from an accumulation of deleterious alleles associated with domestication and a relaxation of selective constraint.

## Discussion

### Intronic and exonic substituion rates

#### Silent and neutral substitutions

Most sites in noncoding regions of the genome in mammals are generally thought to be free from selection and thus both dI and dS are neutral and should accumulate at similar rates [13-15]. In general, we have found that both dI and dS behave in a neutral manner in that they do not vary between genes and accumulate in a clock like fashion. However, dS were consistently higher than dI. A similar result has also been found in primates, with a 60% higher substitution rate found at synonymous than noncoding sites [16]. The authors have argued that their finding was due to an overabundance of CpG dinucleotides, which suffer higher mutation rates due to their methylation by methyltransferases. We confirmed this finding and detected on average a 30% higher rate of silent substitution in coding regions than in noncoding sites and a significantly higher number of substitutions at synonymous sites involving CpG dinucleotides than for noncoding substitutions.

If CpG dinucleotides are ignored, dS and dI are still not identical. Thus, some other force may play a role in reducing substitutions at noncoding sites in some of the genes studied. The noncoding regions in this study include a predominance of sequence adjacent to intron/exon boundaries and 5' and 3'UTRs and some evidence exists for the conservation of selected bases in these regions [17]. The fact that dS does not vary per gene implies that there is no selection for preferred codons in the Bovini tribe for these genes, or that the selection pressure and effective population size for members of the Bovini are so small that dS is in fact behaving neutrally. This confirms previous work [13-15,18] that has found similar silent substitution rates per gene over a wide range of mammals.

### Nonsynonymous substitutions and evolutionary ratios

A number of methods have been developed to identify signatures of selection from the proportion of nonsynonymous to synonymous substitutions per site [19-25]. However, using a simple algorithm described by Nei and Gojorbi [21] we have estimated dN and dS between various lineages in the Bovinae subfamily over dataset encompassing 15 autosomal genes. This algorithm should be accurate as we detected no large bias for transitional to transversional substitutions amongst bovids in our study. Incorporating various lineages from the Bovinae subfamily in the test reduces the requirement of dN/dS ratios to be above one, which is an unrealistic test for selection [12].

Examining dN/dS ratios between bovid lineages has identified the importance of purifying selection during the evolution of the Bovini, which presumably is the case in other lineages, with all of the evolutionary ratios as measured by dN/dS being far below one. Despite the impact of purifying selection, relatively rapid rates of evolution were identified (i.e. high values of dN/(dN+dS)) in the genes 5HT1F, EGF, HFABP, NRIP1 and LACS3. These genes come from a variety of positions in the genomes of the Bovini. Therefore, rapid molecular evolution was not localised to a given region or selection hot spot in the genome and different levels of selective constraint appear to be operating on genes that are separated by relatively small distances.

The fact that the dN:dS ratio in the divergence between species is significantly lower than that detected for within species polymorphism, seems to confirm that positive selection has not driven the evolution of these genes for any of the species or breeds examined.

Members of the Bovini undoubtedly have undergone positive selection due to adaptation to their environments and therefore should show signatures of adaptive molecular evolution due to natural selection. Because genes in this study were selected for their potential role in milk production, we suspected domestic species would show a significantly larger rate of evolution than other species from the Bovini tribe for these genes. However, little difference was identified between taxa, which may be due to the small time over which domestication has occurred and thus the small contribution it has likely made to the differentiation between Bovini genomes. Also the substitutions driven by positive selection appear to be a small proportion of the total, and therefore difficult to detect.

Previous findings have identified a higher accumulation of substitutions in recently diverged species (< 1–2 Mya) when compared to species diverged for longer amounts of time, which may be a result of polymorphisms and deleterious alleles contributing to the rate of short term divergence [26]. A negative relationship between the evolutionary ratio (dN/dS) and time as represented by dS and dI confirms that recently diverged species do in fact harbour a higher proportion of dN to dS, with some of the highest rates for species polymorphism. However, the pairwise comparisons for dN/dS presented in table 6 (lower diagonal) show the highest contributors were between and within breeds of Domestic cow, followed by pairwise comparisons between closely related species and breeds where at least one had a history of domestication (i.e. Domestic cow, Mithan, Yak). This finding does not appear to be repeated between animals separated over similar time frames with no or little history of intensive domestication. Thus, the increase in short term divergence appears to be more pronounced in domestic species.

In a previous study [26] examined protein coding and d-loop sequences from mitochondrial data in avian and primate lineages, while we have examined mutation and substitution in nuclear genes. If any of the estimates in [26] originated from a population that had undergone a serious bottleneck in the past such as humans [27], the effects for these populations may mimic those found for domestication as it usually involves a reduction in effective population size, which leads to a less effective response to selection and an increase in the fixation of deleterious mutants [28-30]. However, many representatives of the Bovini have undergone major population bottlenecks, such as the Bison, which was nearly driven to extinction in the late 1800's [31] and no increase is seen for the rate of short term divergence. However, these population bottlenecks have occurred relatively recently in comparison to humans. Thus, the dN/dS rate is high in the recent evolution of Domestic cattle, Mithan and Yak and remains high in polymorphisms in *B. taurus*. This finding also appears to be true for humans [12], who have gone through an ancient population bottleneck and a relatively recent population expansion, especially when low frequency polymorphisms are not removed.

Evidence suggests that the accumulation of substitutions in recently diverged species typically only applies to mitochondrial DNA [32-34], which is nonrecombining, maternally inherited and thus has a smaller effective population size than genomic DNA. Therefore, deleterious alleles are an important component of DNA polymorphism for mitochondrial sequence, these deleterious mutations appear to accumulate as polymorphisms, but do not contribute to species divergence due to negative selection [35]. To our knowledge the finding of high dN/dS between recently diverged species has never been reported for nuclear DNA. However, we have identified a similar phenomenon for domesticated animals at nuclear genes, with a high evolutionary rate for polymorphisms between breeds of domestic cow and an increased rate of evolution between species for pairwise comparisons where at least one domestic species was examined. This finding is most likely associated with the reduction of selective constraint and effective population size in domestic animals that has decreased the efficacy of negative selection and increased the rate of polymorphism and fixation of deleterious alleles. Previous studies in domestic rice have found an increase in the accumulation of deleterious alleles [30], and this also appears to be the case in cattle. Therefore, these findings may have important consequences to anyone attempting to find signatures of positive selection using a McDonald-Kreitman test from mitochondrial DNA or tests using genomic polymorphism data from domestic species. Therefore, the McDonald-Kreitman test is probably not an appropriate test to identify positive selection in domestic species because of the relaxed selective constraint that is associated with domestication. It also appears that deleterious alleles can exist in a domestic population at a high frequency without being fixed for quite some time, and removing rare polymorphisms will not automatically overcome this problem if the effective population size is small. Therefore care should be taken when inferring the neutral rate of mutation from polymorphism data in some species.

### Ancient polymorphisms and lineage sorting, an alternative to detecting rapid evolution from polymorphism data

Lineage sorting in closely related species is not an uncommon observation [36-38]. In the present study a number of ancient polymorphisms in the coding regions of genes were used to infer the neutral rate of evolution (dN/dS = 0.065). These substitutions most likely were polymorphic in the ancestral population from which they were derived, and in the absence of selection the probability of their loss and fixation in future lineages is governed by the neutral theory of molecular evolution [39]. Therefore, if the evolution of the majority of sites in the Bovini has been neutral the ratio of ancestral amino-acid to synonymous polymorphism should be equal to the ratio in divergence between any given species. Likewise, if selective constraint has been reduced within a species a significantly higher ratio in recent species polymorphism than in ancient polymorphism should be seen. Overall a significantly higher rate of evolution in a number of pairwise comparisons involving Mithan was detected with significant or nearly significant differences detected between comparisons of Mithan and Domestic cattle, Banteng and Gaur. Therefore, it appears that there has been a relaxation of selective constraint in the lineage leading to Mithan. We have also confirmed previous findings by detecting relaxed selective constraint in some breeds of *B. taurus*, with a significantly higher ratio of nonsynonymous to synonymous polymorphisms between Hereford and Tuli when compared to the ratio in ancient polymorphisms and a nearly significant difference detected between some breeds of buffalo. This confirms our previous findings and suggests that intensive domestication and strong artificial selection decreased selective constraint and increased the number of amino acid changing polymorphisms in Domestic cattle.

### Conclusion

In conclusion, the rate of amino acid change (dN) and the evolutionary ratio (dN/dS) was most influenced by the gene and the exon sampled. Of the amino acid changing mutations examined the majority were conservative mutations and the ratio of radical to conservative mutations was not significantly different for any of the variables tested due to the relatively small number of radical mutations. Different genes and regions within a gene may experience different rates of mutation due to their roles, expression patterns, position in the genome or protein and differing levels of selective constraint which in turn effects the rate of evolution, presumably due to differences in the level of selective constraint. Conversely, as species diverge the small number of radical mutations may be explained by the conservation of physiochemical properties, which are often conserved to maintain protein function even across evolutionary distant species [40].

Comparing the rate of polymorphism between breeds of *B. taurus *and *B. bubalis *identified a higher accumulation of nonsynonymous to synonymous mutation in *B. taurus*. Mutation rates are thought to be elevated on smaller time scales (i.e. within species) when compared to greater evolutionary distances between species as deleterious mutations can exist at moderate frequencies within species. This phenomenon appears to be amplified in domestic cattle. Therefore, tests that rely on within species polymorphism as a proxy for the neutral rate of evolution, like the McDonald-Kreitman test, may not be suitable for domestic animals. Interestingly, the presence of ancient polymorphisms could be useful for detecting signatures of selection at the molecular level. In that tests that mimic the McDonald-Kreitman test by using ancestral polymorphism to infer the neutral mutation rate may be more useful for identifying rapid rates of molecular evolution between closely related species. We have found that a greater rate of nonsynonymous substitutions between domestic and nondomestic species separated over similar time frames. The rate of evolution when compared to the neutral rate in polymorphism was not significant, however, in ancient polymorphisms we identified high evolutionary rates for dN/dS between domestic species for divergence especially in Mithan and within Domestic cattle. This result implies that domestication reduces the ability of animals to remove unfavourable mutations from the population as they have very low N_e _and are subject to strong artificial selection, which may have a role in unintentionally increasing the number of deleterious mutations. We believe this phenomenon will be common in the coding sequences of domestic cattle and should not be dependent on the methodology used to anlalyse molecular evolution. However, future work may look at applying codon based models such as those implemented in PAML to identify individual sites that have been subject to positive selection, which have undoubtedly occurred in at least some genes during domestication. Although differentiating between positive selection and reduced selective constraint may be difficult.

Finally, we have also identified a significant difference in the rate of substitution at synonymous and noncoding sites in the Bovinae that is that is partially, but not completely, explained by CpG dinucleotide frequency differences. In particular, we have found that the number of noncoding substitutions per site (dI) was significantly smaller than the silent substitution rate dS, which appears to be the result of a high proportion of CpG dinucleotides at synonymous sites inflating the mutation rate and this feature may have important consequences for estimating divergence times between species at these sites.

## Methods

### Genes, species, breeds and animals

Candidate genes were selected using *a priori *knowledge of the genes position in relation to suspected milk QTL, or evidence of higher than average rates of molecular evolution for pairwise comparisons between *Bos taurus*, *Homo sapiens *and *Bos indicus *[12,41]. In total 84 amplicons, which typically consisted of exonic and flanking noncoding regions, were sequenced from the following 15 genes: NRIP-1, PIT-1, ITGBP-5, 5-HT-1F, IGFBP-2, IGFBP-5, H-FABP, LACS3, 5-HT-6, GMEB-1, EGF, ERA, PRRP, PABPC-1 and MFGE8, in at least 1 individual from genomic DNA. DNA was extracted from the following breeds and species of cattle: 1 × Holstein (*B. taurus*), 1 × Tuli (*B. taurus*), 1 × Hereford (*B. taurus*), 2 × Banteng (*B. javanicus*), 2 × Gaur (*B. gaurus*), 2 × Yak (*B. grunniens*), 1 × Mithan (*B. frontalis*), 2 × Bison (*Bison bison*), 1 × Murrah buffalo (*Bubalus bubalis*), 1 × possible hybrid River/Swamp buffalo (*Bubalus bubalis/carabensis*), 1 × Swamp buffalo (*Bubalus carabanesis*), 2 × Cape buffalo (*Syncerus caffar*), 2 × Eland (*Taurotragus oryx*) and 3 × nondescript breed of Indian buffalo (*Bubalus bubalis*). Complete information on these samples and sequences is detailed in MacEachern et al [3]. Polymorphism data was collected using between breed/subspecies information for Water buffalo and Domestic cattle. Polymorphism data was also obtained within breeds using an additional 8 unrelated Holstein bulls, which were sourced from Genetics Australia.

### Sequence analysis

Consensus and ancestral sequences were generated using computer modules written in PYTHON, which have been described in detail [3]. For each amplicon the consensus sequences from each representative of the Bovinae subfamily were aligned. Pipelines were developed to undertake pairwise comparisons between all samples to count the number intronic/noncoding substitutions (Ki), nonsynonymous substitutions (Ka), synonymous substitutions (Ks), intronic substitutions per site (dI), nonsynonymous substitutions per site (dN), synonymous substitutions per site (dS) and the number of radical (Dr) and conservative (Dc) substitutions. Exonic sequences and the corresponding open reading frame (ORF) were identified by cross-referencing alignments from known Bovine and Human protein coding genes [12]. All information per amplicon was summed up per gene; therefore, information has been collected for each exon, intron and gene for all species successfully sequenced. Polymorphism information was retrieved from pairwise comparisons amongst breeds of *B. taurus *and *B. bubalis *and within breeds of *B. taurus *for dI, dN, dS, Dr and Dc. To reduce the chance of sequencing errors infiltrating our dataset, polymorphism data from heterozygous individuals was ignored unless both alleles were also found in homozygous form; alternatively the most frequent allele was represented at that site.

### Statistical analysis

#### Phylogenetic reconstruction

Phylogenetic trees were generated using the neighbour joining algorithm and Kimura's two-parameter distance using nonparametric bootstrapping with 5,000 replicates from 21,055 base pairs of intronic sequence and 1,526 segregating sites in the MEGA4 package [15].

#### Detecting selection in the bovine lineage

Each species was compared to the inferred ancestral sequence to determine whether various lineages had evolved more rapidly than others. Thus, dN and dS were calculated for each lineage in the phylogeny and internode divergence was compared between all lineages in the tree. Evolutionary ratios varied greatly between genes and amplicons often from 0 to infinity. Therefore, evolutionary ratios were calculated using the ratio dN/(dN+dS) and Dr/(Dr+Dc) per exon and per gene.

Differences were examined using general linear models (GLM) in GenStat for Windows 8^th ^Edition. The first model tested (GLM 1) was used to detect whether certain amplicons or genes had evolved rapidly in certain species since their divergence from the ancestral sequence. The model tested was (1) **y = μ + g + a + s + e **where *y *= dN, dS, dI, dN/(dN+dS) and Dr/(Dr+Dc), μ = the mean effect, g = the effect for each gene, a = the effect of each amplicon within gene, s = the effect of each species and e = the residual error. An F-test for gene was completed using the mean square for gene divided by the mean square for amplicon within gene to determine if gene effects were significant above those detected for amplicon within gene.

Thus, if positive selection has driven the divergence in Domestic cattle for some or all of the genes sequenced, a significant effect for species should be seen, with the lineage leading to *Bos taurus *having significantly higher evolutionary rates when compared to other species. Typically positive selection is inferred if dN/dS ratios are greater than one. However, this is a conservative test for selection, comparing dN and dS amongst closely replated species tests neutrality by examining deviations within the phylogeny for the dN/dS ratio. Any deviations in dN/dS would violate the strictly neutral model [42].

The second model (GLM 2) fitted the interaction between gene and species to determine if particular species and genes were responsible for the differences in the rates of evolution detected in GLM 1. The model fitted was (2) **y = μ + g + s + g.s + e **where the interaction g.s tests the difference between gene and species for rates of evolution. Therefore, a significant interaction between (g.s) should be detected if the differences between species vary from one gene to the next. This may be evidence of positive selection driving gene evolution in a particular species.

#### Comparison of synonymous and intronic sites

If selection operates on synonymous but not intronic substitutions a lower substitution rate at silent sites in exons compared to substitutions at intronic sites would be expected. An increase in the proportion of substitutions at synonymous sites involving CpG dinucleotides when compared to substitutions at noncoding sites has been reported to increase dS in primate genomes [16]. To determine if this phenomenon was present in genomes from the Bovini we examined substitutions at synonymous and noncoding sites for differences in the relative number of substitutions involving CpG dinucleotides. CpG dinucleotides were identified as a section of DNA where a cytosine then a guanine nucleotide occurs adjacent to each other on the same DNA strand. CpG dinucleotides are likely to mutate to TpG or CpA [16]. Hence, any substitution in the alignment involving a synonymous or noncoding site was determined to either involve a CpG dinucleotide or not. A comparison of the number of substitutions per site involving CpG dinucleotides at synonymous and noncoding sites, such as those described above, may explain any differences between substitution rates at both sites. Substitutions were also compared between noncoding and synonymous sites for non-CpG related sites to determine if CpG related substitutions were responsible for all the variation between sites.

#### Rate variation and relationships between substitution rates

Examining the relationships between various substitution rates should be a quick reference to gauge the accuracy of methods applied to estimate substitution and evolutionary ratios. Therefore, the relationships between substitution rates at different sites were compared by examining the total numbers of substitutions that have accumulated since the divergence from the ancestral sequence across all 15 genes for each species. Examining the relationship between synonymous and noncoding substitutions per site should show a strong correlation if substitutions at both sites are neutral, and the relationship these neutral sites have with the nonsynonymous substitutions and the evolutionary ratio estimated across all genes (dN/dS) should highlight how dN and dN/dS change over increasing evolutionary time.

#### Testing for selection or reduced selective constraint in domestic animals

To test for evidence of rapid molecular evolution in domestic animals contingency tables compared the ratio of nonsynonymous mutations to synonymous mutations. Comparisons were between an ancestral sequence and a domesticated species with a closely related species that has not been domesticated. If the observed substitutions between the ancient and domestic animals are neutral the ratio of nonsynonymous to synonymous fixed differences should be the same for domestic and wild animals when compared to the ancestral sequence. Within species variation was also examined between *B. taurus *and *B. bubalis*. If domestication has increased the rate of deleterious mutations in *B. taurus *the ratio of nonsynonymous to synonymous polymorphisms should be higher for comparisons within *B. taurus *than for comparisons within *B. bubalis*.

#### Comparing inter-specific divergence and intra-specific polymorphism

Differences between lineages for the dN/dS ratio are deviations from neutral theory. However they may be caused by positive selection or relaxed selective constraint, possibly as a result of the domestication of *B. taurus*. Analysing species divergence and within species polymorphisms should differentiate between the two. Therefore, evolutionary rates in divergence for pairwise comparisons between each species and the Hereford genomic sequence and the within species polymorphisms detected in 8 Holstein samples were compared using contingency tests. Analysing pairwise comparisons between breeds of *B*. *taurus *with the polymorphism data collected within Holstein animals should allow us to detect whether positive selection was responsible for driving the evolution in the coding sequence for these genes during their domestication.

#### Ancestral polymorphisms

The phylogenetic relationship of these samples identified a number of variable sites that were abnormally inherited within the Bovini tribe [3]. These aberrant sites appear to have remained polymorphic for many generations and are probably neutral or nearly neutral. Therefore, Ka and Ks at these sites were compared to Ka and Ks found between and within species using a modified McDonald-Kreitman test [2] to identify possible signs of positive selection.

## Competing interests

The authors declare that they have no competing interests.

## Authors' contributions

SM designed primers, collected samples, completed all sequencing and analysis and prepared the manuscript for submission. AM and AMc contributed ideas and helped with computer analyses. KS contributed to the candidate gene study. JM and MG helped coordinate the study and provided statistical and writing support. All authors read and approved the final manuscript.
